# Supplementary figures and images for: Evolution of sensory systems underlies the emergence of predatory feeding behaviours in nematodes
Source: bioRxiv. 2025 May 21:2025.03.24.644997. Originally published 2025 Mar 25. Preprint. [Version 2] doi: 10.1101/2025.03.24.644997 (PMC11974876; doi:10.1101/2025.03.24.644997)

Figure S1

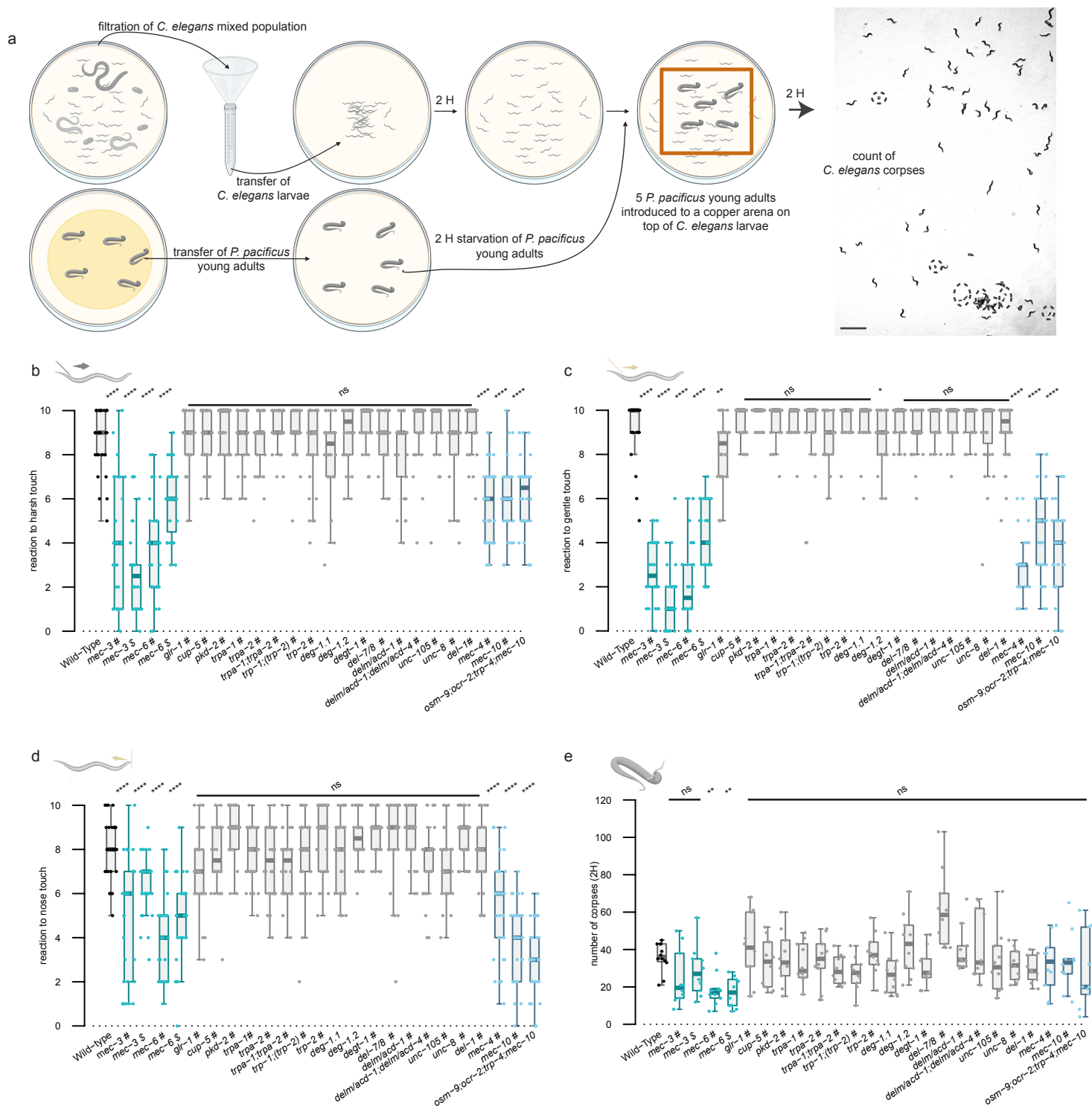

a

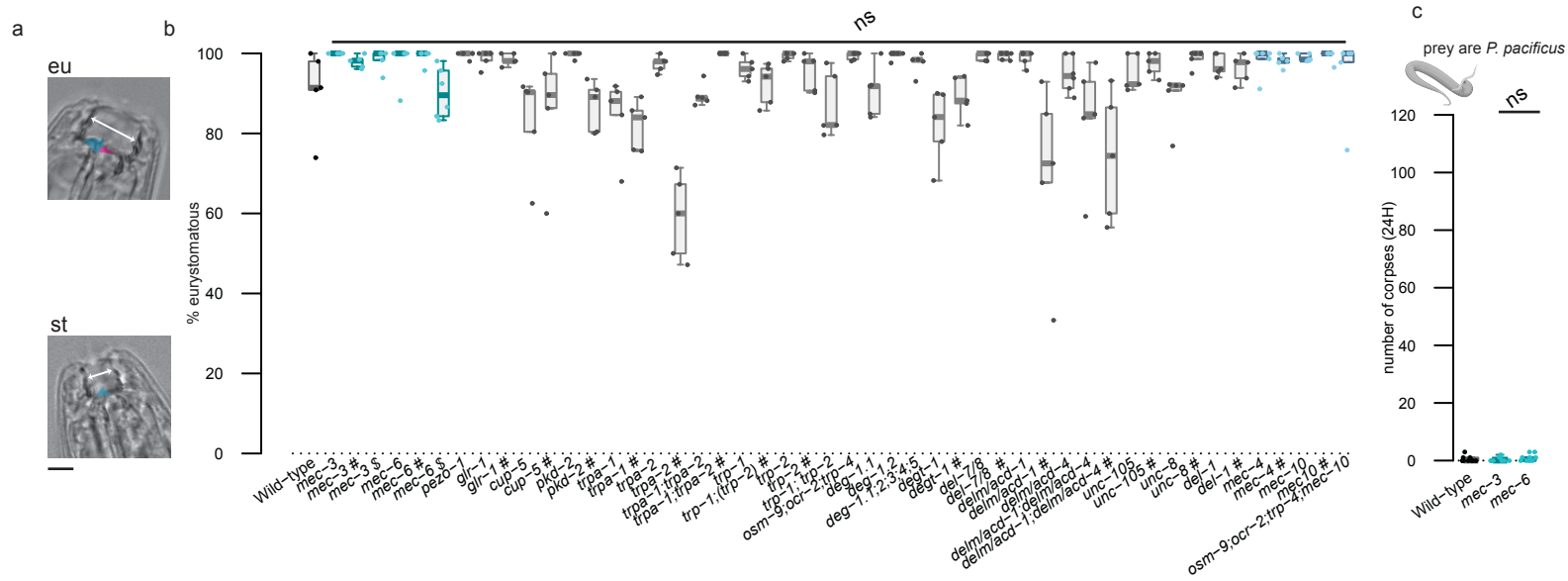

Figure S3

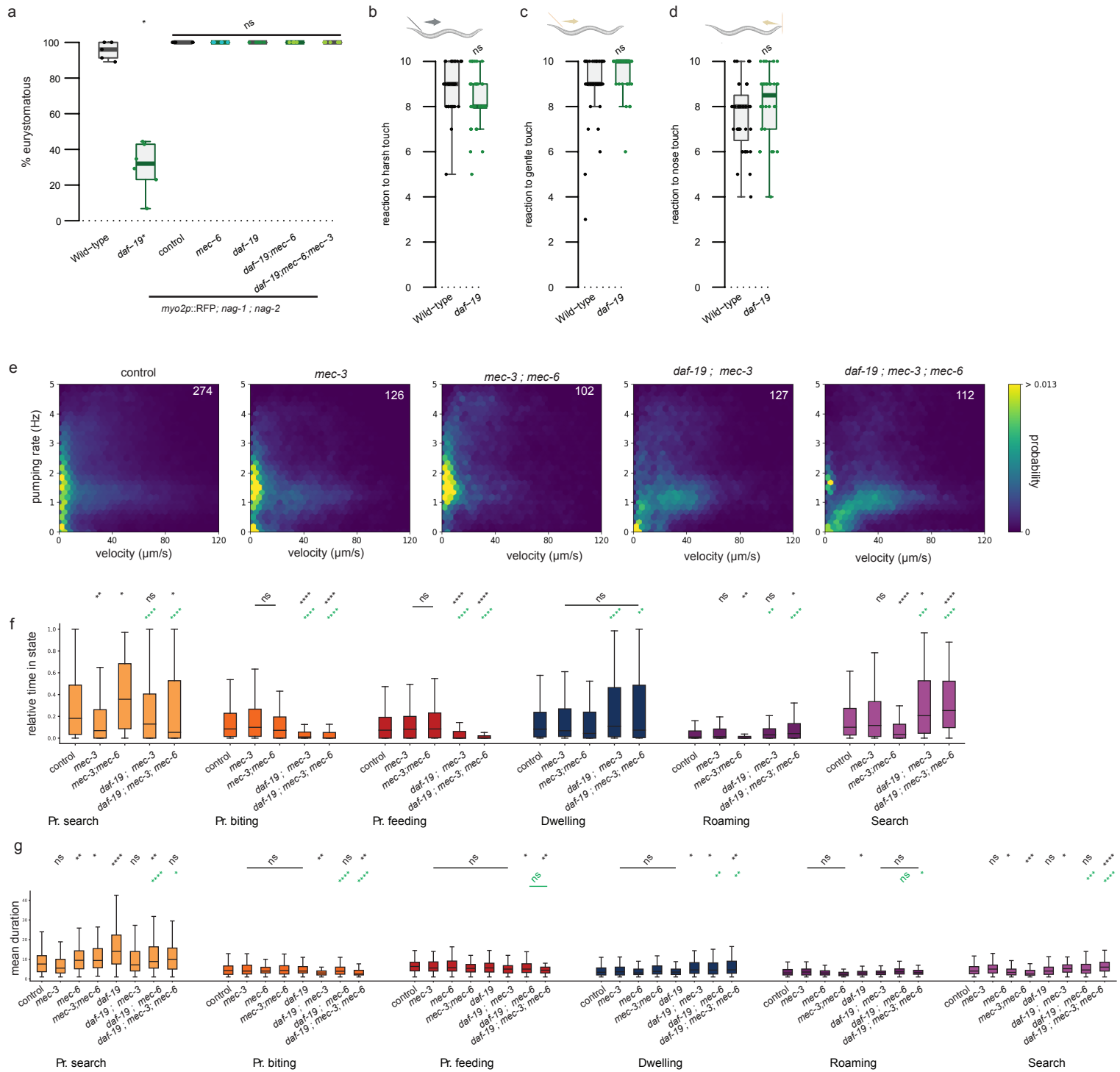

Figure S4

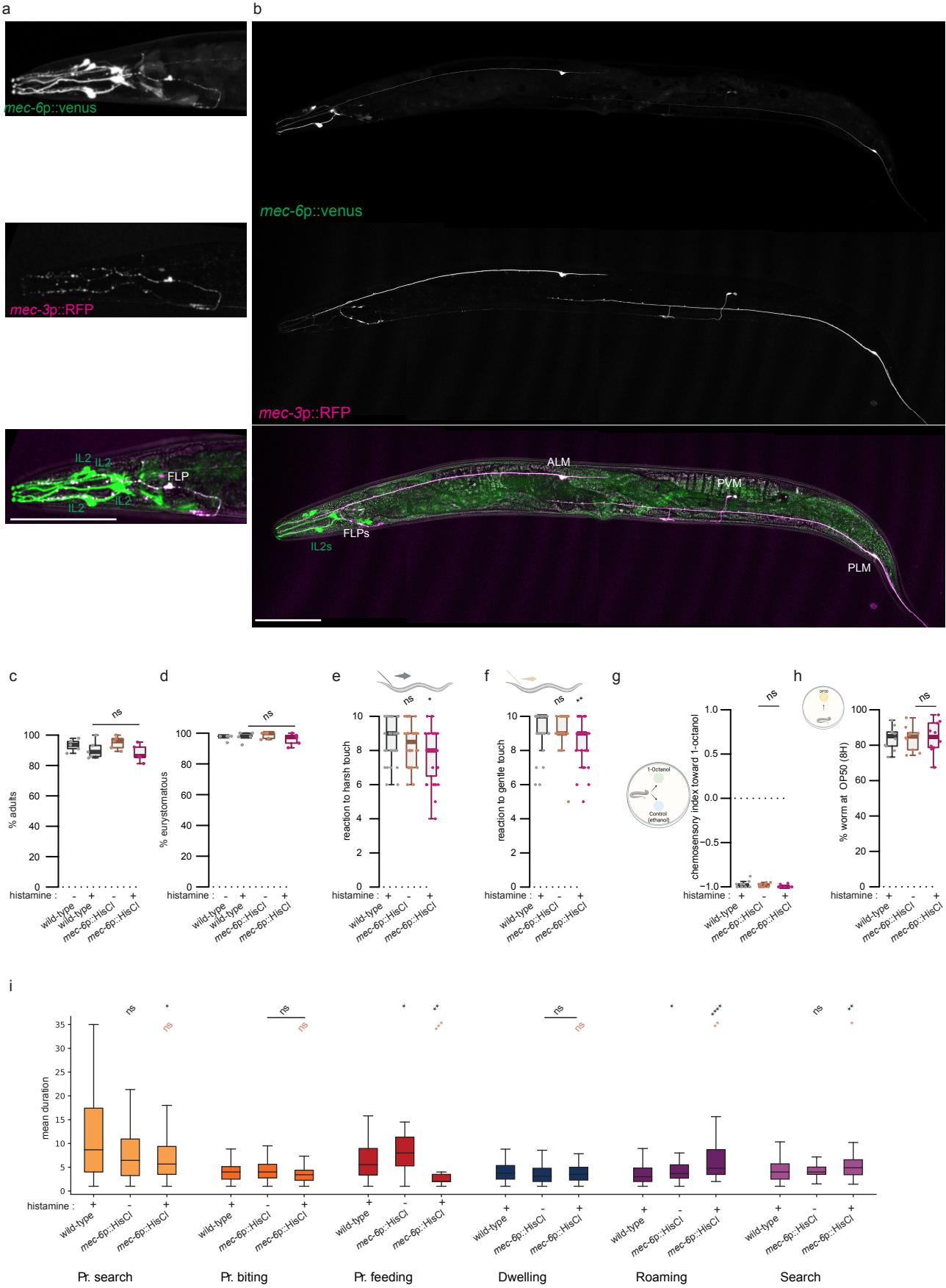

Supplement: Supplement 1 — Supplementary Figure 1: Mechanosensation and predation in P. pacificus (A) Schematic of the corpse assay protocol. Five starved P. pacificus predators of the strain of interest are introduce in an area with an abundancy of C. elegans larvae. After 2 h, predatory success is assessed by counting the number of larval corpses inside the arena. In the image corpses can be observed inside the circles. Scale bar is 500 μm. (B) Mechanosensory assays to harsh touch, (C) gentle touch, and (D) nose touch. Each assessment is the result of ten consecutive trials of each worm. At least 30 worms were tested per strain. (E) Number of C. elegans corpses counted after two hours of contact with the indicated P. pacificus strains as predator. At least 10 assays were performed. Statistical tests: one direction Wilcoxon Mann Whitney with Benjamini-Hochberg correction, non-significant (ns), p-value ≤ 0.05 (*), ≤ 0.01 (**), ≤ 0.001 (***), ≤ 0.0001 (****). Schematics were made with biorender. Supplementary Figure 2: Mechanosensation does not affect developmental plasticity or kin-recognition (A) P. pacificus is developmentally plastic leading to one of two mouth forms, stenotomatous mouth (st, bottom) with the dorsal tooth (blue) or eurytomatous (eu, top) with an additional subventral tooth (red) and a wider opening. Scale bar is 10 μm. (B) Percentage of eu was assess 5 time for each strain. (C) Number of wild-type P. pacificus corpses counted after 24 hours of contact with the indicated P. pacificus strains as predator. Statistical tests: one direction Wilcoxon Mann Whitney with Benjamini-Hochberg correction, non-significant (ns), p-value ≤ 0.05 (*), ≤ 0.01 (**), ≤ 0.001 (***), ≤ 0.0001 (****). Schematics were made with biorender. Supplementary Figure 3: Behavioural analysis of sensory deficient worms (A) Percentage of eu morphs were assessed 5 time for each strain. As shown in Moreno et al 2019, mutations in Ppa-daf-19 lead to a higher occurrence of st mouth form which is overcome by incl [file media-1.pdf]
